# Mothers’ perception of maternal and child health information disseminated via different modes of ICT in Nigeria

**DOI:** 10.1111/hir.12235

**Published:** 2018-09-24

**Authors:** Oluwaseun I. Obasola, Iyabo M. Mabawonku

**Affiliations:** ^1^ E. Latunde Odeku Medical Library College of Medicine University of Ibadan Ibadan Oyo Nigeria; ^2^ University of Ibadan Faculty of Education Ibadan Oyo Nigeria

**Keywords:** Africa, west: consumer health information, eHealth, focus groups, health literacy, midwifery, questionnaires, women's health

## Abstract

**Background:**

A few studies have examined mothers’ perception of ICT and maternal and child health (MCH) information promoted using ICT. The effectiveness of different modes of delivery of such information is unclear.

**Objective:**

To investigate mothers’ perceived usefulness of ICT and MCH information disseminated through e‐health projects in Nigeria.

**Methods:**

The study was a descriptive survey that was based on the mixed method paradigm. A questionnaire was used to collect data from 1001 mothers involved in ongoing ICT based projects in Nigeria. The mothers were selected using a convenience sampling technique. Four focus group discussion sessions were also organised for thirty mothers.

**Results:**

Mobile phones were viewed as useful (35.0%) or very useful (42.2%) and radio as useful (34.8%) or very useful (57.5%%). But they expressed a negative perception towards the use of DVD/TV (Not useful, 66.5%) and the Internet/computer (Not useful 67.7%). Mothers’ perception of MCH information disseminated was also positive. They reported the need for more MCH information products in local languages using acceptable ICT.

**Conclusion:**

Mothers’ perception of preferred modes of delivery of maternal and child health information varies according to location.

**Key Messages**

- Mobile phones are useful and acceptable tools for improving mothers’ access to MCH information.
- The use of at least two different channels, for example, radio and mobile phone, is important, particularly, when Internet access may be less accessible.
- The preference for modes of communication about maternal and child health information (and languages of presentation) may vary considerably across different communities.

## Background

The literature indicates that prior to the advent of information and communication technology (ICT), the dissemination of maternal health information as a strategy for preventing pregnancy and birth complications was based on print and oral communication approaches, for example, antenatal classes. This traditional approach seems to have little impact, as studies have indicated little effect of these information methods on health outcomes (Ferguson, Davis & Browne, 2013; Gagnon & Sandall, 2007).

To improve maternal health literacy and outcomes, the World Health Organisation‐WHO (2016) and Federal Ministry of Health in Nigeria (2011) recommend scaling up the dissemination of maternal and child health (MCH) information to mothers to improve health outcomes in the developing countries, especially in Nigeria. Some authors (Anya, Hydara & Jaiteh, 2008; Doctor, Findley, Cometto & Afenyadu, 2013; Parmar, 2010) have also highlighted the need to improve information dissemination, as some pregnancy complications and birth outcomes experienced in developing countries have been attributed to inadequate MCH information dissemination and limited access to health services. The need to improve maternal health and birth outcome has led to the adoption of ICT for MCH information dissemination to encourage the adoption of safe MCH practices disseminated by health workers (Agency for Health Care Research Quality, 2012; Edwards, 1995). The preference for the adoption of ICT in scaling up MCH services has grown widely across sub‐Saharan Africa. Nigeria too is gradually adopting ICT in public health facilities for MCH care (Oyeyemi, 2012; Victoria, 2015).

To maximise the gains of ICT for promoting information on safe MCH practices in Nigeria, it is important to explore the mothers’ perception of ICT and information products from the channel. This is because earlier studies have linked negative perceptions by mothers (Castle, Thompson & Karlyn, 2011; iied, 1992; Parkkola, 2006a,b) to unwillingness to use ICT to receive MCH information which affected their participation in ICT based projects. These studies indicate that neglecting the perception of the target group when designing ICT based projects may affect the acceptance of ICT, which will discourage the adoption of MCH information promoted through ICT. The varying perceptions of mothers regarding MCH information products and ICT use need to be addressed to ensure the effectiveness of ICT based projects (Parkkola, 2006a). Focusing on mothers’ perception could reveal more information that could be central to the design of MCH information products and ICT based projects.

Most studies (Fajembola, 2011; Oyeyemi, 2012) on the use of ICT for MCH information have focused on the influence on deliveries and referrals, with negligible attention paid to mothers’ perception of ICT and the MCH information disseminated. This is a major gap in the literature. Therefore, investigating mothers’ perception of ICT and MCH information disseminated in Nigeria becomes pertinent.

As a follow‐up to a previous study (Obasola & Mabawonku, 2017), this study focused on perception of ICT disseminated MCH information accessed by women in our previous study. The current study examined mothers’ perception of the use of television, radio, mobile phones and the Internet, as well as MCH information disseminated when implementing ICT based projects for MCH in Nigeria. This is to improve the effectiveness of ICT based projects and to provide information which would be useful in developing MCH information products.

## Mothers’ perception of ICT used for MCH information dissemination

Studies accessing mothers’ perception of the usefulness of ICT and MCH information disseminated through the channel are rare. Intention based theories, such as the Unified Theory of Acceptance and Use of Technology (UTAUT), clearly stress the importance of the perceived usefulness, ease of use, social influence, facilitating conditions and control factors as some of the major factors to consider when planning ICT based programmes (Venkatesh, Morris, Davis & Davis, 2003). The UTAUT illustrates how the perceived usefulness and the ease of use of ICT could have profound influence on the adoption of an information system. The UTAUT is the outcome of the harmonisation of eight models that authors had used to explain information systems usage (Ajzen, 1991; Ajzen & Fishbein, 1980; Bandura, 1988; Davis, 1989; Davis, Bagozzi & Warshaw, 1989; Rogers, 2003). The UTAUT model is frequently applied in studies on user acceptance of technology.

Kijsanayotin, Pannarunothai and Speedie (2009) were among the first set of scholars to adapt UTAUT to predict the use of ICT for administrative purposes when implementing e‐health projects in Thailand. The results of the study show that if users perceive ICT services as useful, they will choose to use them. Therefore, willingness to use ICT as a source of health information may be dependent on mothers’ perception of the channel. As a result, expression of interest in the form of demand could be equated with positive perception of ICT by mothers. This can have significant influence on the adoption of ICT tools.

Parkkola (2006a,b) in a qualitative study on mothers’ views when designing ICT solutions observed a positive disposition by mothers towards ICT once they were convinced of its benefits. It was also reported that the demand and attitude to use ICT by mothers were closely related. The study indicated that the motivation to use ICT was based on the needs and the characteristics of available ICT. This ultimately affected the use of ICT by mothers to access health information.

In the same vein, Declercq, Sakal, Corr and Applebaum (2006) note that pregnant women in the United States rely primarily on the Internet for their health information needs. The 4‐year survey revealed that about two‐thirds (64%) of the respondents (pregnant women) enjoyed using a smartphone to access health information, and 82% had gone online using a computer system. Some of the women reported using tablet devices (35%), mobile phones (33%) and iPad touch devices (21%) to access online health information. The authors argue that, since the use of the Internet among women in the United States is high; it should be integrated with birth education classes.

Similarly, an evaluation of an ICT based project in Nigeria (Gombe State) indicated that 50% of the women involved in the project were happy to receive MCH information through mobile phones (Society For Family, 2012), as over 24 000 calls were received within four months of the project initiation, and about 400 women provided positive feedback on the quality of information and support from the call centre. However, the study indicated that the use of ICT may be affected by the level of IT skills possessed by women. Likewise, a study by Balogun et al. (2012) involving 399 mothers, at a tertiary health clinic in Lagos, Nigeria, reported that mothers (77%) were positively disposed to use mobile phones for receiving appointment reminders. Brown, Oluwatosin and Ogundeji (2015) also observed a related trend in Oyo State, Nigeria, where 95.1% of the 614 mothers expressed willingness to use their cell phones to receive appointment reminders. Only 4% of the mothers were not willing to use their mobile phones to receive information on immunisation.

The positive attitude observed in these studies differed from the findings of (Parkkola, 2006b). The author reported a negative disposition to ICT by mothers in Finland because of limited knowledge on the use of ICT. A study in Kaduna State, Nigeria (Castle et al., 2011) also revealed a negative disposition owing to low ICT skills by mothers using mobile phones. As a result, most of the mothers lacked willingness to participate in the ICT based projects. These results further underscored the need to pay attention to mothers’ views on the type of ICT tool and the need to train mothers on the use of ICT tools when promoting health messages, as perception may vary from one setting to the other.

In spite of the growing body of the literature (Balogun et al., 2012; Castle et al., 2011) on the acceptance of recent ICT channels as sources of MCH information, studies have shown that women still prefer health tips from radio and television (O'Mara, Babacan & Borland, 2010; Parmar, 2010). These studies have shown that the available ICT channel may not be the most preferred source of MCH information. Likewise, the more recent source may not be the most used, because women in the developing countries are positively disposed to use old ICT, such as the radio and television. Therefore, there is the need to be cautious in the deployment of technology for communicating MCH information. It is important to investigate the mothers’ perceptions of ICT tools and the information being transmitted when evolving ICT based projects. The acceptance of the ICT can depend on the effectiveness of ICT based projects.

## Mothers’ perception of ICT disseminated MCH information

Several studies have investigated mothers’ perception of health information disseminated through ICT. One of such studies is a systematic review by Sayakhot and Carolan‐Olah (2016) which examined the use of the Internet by pregnant women in Mexico, Italy, China, Turkey, Sweden, United States of America and the United Kingdom. The authors found that, in six out of the seven countries, a significant proportion (80% to over 90%) of women sought MCH information frequently from the Internet. It was only in Turkey that 44% of the women reported obtaining information during pregnancy once or twice a week. Most of the women sought information on a number of topics, such as foetal development, nutrition and stages of birth. They also found that women involved in four out of the seven studies found health information on the Internet to be reliable and useful, as this influenced their pregnancy related decisions. Thus, the majority of the women (182–1347) who participated in the studies reviewed were active users of online pregnancy related information, because they believed the information they accessed was beneficial, enabling them to make informed decision about their health. However, the review did not indicate any influence of online information accessed on health outcomes. The studies reviewed were quantitative in nature; they did not provide an opportunity for disclosure among the study participants to validate data from the surveys.

A survey in the United States by Childbirth Connection (2012) explored pregnant women's use of online health information. The nationwide study with a sample size of 2400 provided the most extensive data on pregnant women use of online health information. In the study, women (60%) rated pregnancy websites as highly trustworthy sources of pregnancy and birth information. The blogs providing such information were the most widely used (by 78%). Online forums and discussion boards followed (76%). Two‐thirds of the women found online video sites (68%), Facebook (67%) and *Wikipedia* (67%) valuable for pregnancy related information. Most of the women (72%) involved in the study also felt reassured to make appropriate health care decisions after accessing online health information. Some of the women had negative emotions, such as feeling overwhelmed (27%), frightened (17%), frustrated (13%) or confused (11%) by the online information. The survey further confirmed women's willingness to use online health information, as well as its acceptance because it was useful in making informed decisions about their health.

The Short Message Service (SMS) via the mobile phone is another important strategy adopted for disseminating MCH information to mothers (Lenhart, 2010). Broom, Ladley, Rhyne and Halloran (2015) found, in a study in the United States, that low income mothers were happy to receive health tips through SMS. The mothers (86.6%) successfully received health tips over 6 months of period and 66.1% requested for a callback. Over 80% of the mothers liked the messages they received, because they felt the messages were easy to read (89%) and relevant (82%). About 75% of them also shared texts with others. Broom et al. (2015) did an evaluation study with 54 participants; it adopted the mixed method prototype. However, the study was limited because the authors could not conduct a formal analysis of qualitative data, owing to limited responses from the participants. The authors highlighted the need for larger and controlled studies adopting the mixed method design to assess mothers’ perception of ICT disseminated messages.

Cormick et al. (2012), in a qualitative study involving 147 pregnant women in two public health facilities in Argentina, note that pregnant women could benefit from health messages disseminated through mobile phones. This was because the majority of women in the public health facilities had access to and were interested in receiving text messages and calls that provided information and education on MCH. Mechael's (2009) ethnographic report in Ghana likewise showed a high demand for and positive disposition to information disseminated using the mobile phone service, which exceeded expectations and overstretched the capacity of the three operators at the call centres used for the project.

These studies were limited because they did not provide the opportunity for generalisation of the study findings. Mecheal's study may be subjective since it was mainly based only on the author's view and research capacity. Oyeyemi's (2012) case–control study observed that the use of mobile phones for disseminating messages in Nigeria has helped to overcome the challenge of mothers’ access to health workers or facilities, allowing them to access MCH information without having to travel long distance. This was probably the reason for its acceptance by mothers. However, Oyeyemi's study lacked baseline data from study areas to ascertain the influence of mobile phone use on health outcomes.

Regarding the use of radio and television, a survey by Bowen (2010) on MCH programmes among 1809 mothers in Ghana revealed that women who participated in the study trusted health information from television and radio (70%) more than other sources. On the other hand, in Somalia, a qualitative study which involved the use of radio and television for promoting immunisation pointed to the negative perceptions of mothers towards promotion of immunisation. It seems the messages failed to show connections between the vaccines promoted and disease prevention (iied, 1992).

These studies documented evidence on mothers’ perception towards MCH information from ICT. They further indicated the need to consider the perception of the target group of the health messages being disseminated, as perception may vary from one context to the other.

## Research question

What is mothers’ perceived usefulness of ICT and MCH information disseminated using ICT in Nigeria?

## Method

The descriptive survey adopted both quantitative and qualitative approaches to data collection. A questionnaire and focus group discussion (FGD) guide were used to elicit information from mothers involved in ICT based projects in Ondo, Imo, Kaduna and Gombe States in Nigeria. The questionnaire for the study was developed based on cues from the UTAUT scale for measuring perceived usefulness of an information system (Davis, 1989; Venkatesh et al., 2003). The questionnaire was pretested and the reliability coefficient calculated using Cronbach Alpha was 0.86.

Nine public health facilities – Ondo (4), Imo (1), Kaduna (2) and Gombe (2) States – were purposively selected. Convenience sampling was employed to select 1001 mothers who were attending maternity clinics at the health facilities. The quantitative data were complemented with four sessions of focus group discussions in Ondo (10), Imo (8), Kaduna (6) and Gombe (6) States. Research assistants who could speak the local languages were employed for the FGD sessions which were recorded using a mobile device. Data collection, which started in September 2014, took over nine months. Ethical approval was obtained from the University of Ibadan Ethics Committee. Informed consent of the participants was obtained. While the quantitative data were analysed using descriptive statistics, qualitative data were transcribed into English and content‐analysed.

## Results

The results presented in Table 1 indicates that, while the perception of the mothers on mobile phones (Useful, 35%, Very Useful, 42.2%) and radio (Useful 34.8%, Very Useful, 57.5%%) was positive, that of the computer/the Internet (Not Useful 67.7%) was negative. The mothers indicated that phone and radio were the most useful of all ICT tools.

**Table 1:** Perceived usefulness of ICT by mothers

Perceived usefulness of ICT	Responses	Ondo	Kaduna	Imo	Gombe	Total	%
Radio	Not useful	55	8	5	3	71	7.6
	Useful	124	57	51	92	324	34.8
	Very useful	221	333	92	190	536	57.5
DVD/TV	Not useful	219	62	88	250	619	66.5
	Useful	139	32	55	29	255	27.3
	Very useful	42	4	5	6	57	6.1
The Internet/Computer	Not useful	226	58	135	212	631	67.7
	Useful	152	12	34	71	271	29.1
	Very useful	22	4	1	2	29	3.1
Phone	Not useful	101	41	20	50	212	22.7
	Useful	178	41	34	73	326	35.0
	Very useful	121	16	94	162	393	42.2

N = 931 Useful/Very Useful = positive, Not Useful = Negative.

Results from the FGD sessions with the mothers in Imo State revealed a positive perception towards the Internet. For example, a mother from Imo State noted the usefulness of the Internet:

I actually get more information from the Internet. If I am not satisfied with the information from the television or radio, I check different websites until I'm satisfied. This is not possible with the television or radio. This is why I prefer the Internet.

Other mothers in Imo State were also of the view that the Internet was an important source of MCH information and that it provided the opportunity for them to access health information at their convenience.

The view of the mothers during FGD in Ondo, Gombe and Kaduna States was different, and women in the three states indicated a positive preference for mobile phones because of its relevance in meeting specific health information needs (especially during emergencies). For example, a mother in Ondo State asserted thus:

I prefer the mobile phone. Once I call the matron, I always get immediate answers to pressing concerns about my health. This has helped me to stay healthy. This helped in saving my life and that of my unborn child at the early state of the pregnancy when I was bleeding.

In Kaduna State, a mother noted thus:

I like the mobile phone because I can easily reach Mama Matron (Nurse) anytime. I receive information on pregnancy and child care from the health facility I'm registered through phone calls.

A mother in Gombe State also averred that:

My impression when we were given the phone was good. Many of us could not operate the phone but we were taught how to use it. I use it to call the nurse at the health centre. The nurses can call or sometimes send information to me to come for check‐up.

The quantitative data revealed improved knowledge about health issues surrounding pregnancy and childbirth (97.3%). The mothers were of the view that the use of ICT disseminated MCH information helped them to stay healthy in pregnancy (94.6%). See Table 2.

**Table 2:** Mothers’ perception of the usefulness of MCH information disseminated using ICT

Mother's perception of the usefulness of MCH information from ICT channel	Agree freq %		Disagree freq %	
ICT‐disseminated MCH information has improved my knowledge about health issues surrounding pregnancy and child birth	906	97.3	25	2.7
Over all use of ICT‐disseminated MCH information has helped me to stay healthy in pregnancy	881	94.6	50	5.4
ICT‐disseminated health information has improved my MCH practices (Breastfeeding habit, family planning methods, ANC visits, immunisation etc.)	894	96	37	3.9
ICT‐disseminated health information has improved my use of health facilities	89	95.9	38	4.0
ICT‐disseminated health information has helped me to manage common health conditions in newborns and children	882	94.8	49	5.3
ICT‐disseminated health information has improved my health‐seeking behaviour (knowing what to do and where to get help when in need of health care)	887	95.3	44	4.7
ICT‐disseminated health information has improved decision making about my health	893	95.9	38	4.0
ICT‐disseminated health information has helped me to manage common health conditions (cold, malaria, and diarrhea) observed in pregnancy	87	93.8	58	6.2

N = 931.

The findings in Table 2 were slightly different from the mothers’ responses during the FGD sessions. Some mothers were of the view that, with the exception of interactive radio or television programmes that incorporate feedback through phone calls, MCH information from radio and television was usually limited. The respondents felt that these tools were limited because of the time frame. So, the information provided might not be sufficient. They were of the view that presenting MCH information in different local languages may be better. One of the mothers in Gombe State affirmed this:

When I watch TV programmes, the health information I get is usually limited. They are usually in a hurry because of airtime. It will be better to have more interactive TV or radio programmes in different dialects. This will provide us (pregnant women) more opportunities to clarify issues about our health.

Another mother in Imo State argued that:

The information I get from the television most of the time is usually in English. How I wish they (health workers) could make the information available in other local languages. They should provide information in Ijaw or Urhobo.

The FGD participants in Imo State also stated that the content of the messages from television, radio and phones was not adequate enough when compared with the MCH information they accessed from the Internet.

The sampled mothers indicated that some health issues were not adequately covered by health workers when communicating MCH information through ICT. The issues included dieting and weight control, types of medication to take during pregnancy, how to calculate expected delivery date, postpartum depression, and how to respond to complications such as vomiting and swollen legs in pregnancy.

## Discussion

The results showed that mothers’ perception of MCH information was largely positive. The respondents’ disposition to MCH information received from ICT revealed that they enjoyed keeping up with health tips concerning pregnancy and childcare from these platforms. Some of the mothers reported that the MCH information received from ICT channels was usually adequate, meeting most of their MCH information needs. They were of the opinion that they were more informed about issues relating to their well‐being because of the use of ICT for disseminating information on MCH (See Table 2). This implies that the mothers’ perception of ICT disseminated MCH information was positive. This result is in line with studies (Brown et al., 2015; Childbirth Connection, 2012) conducted in the United States and Africa. These studies indicated positive perception to MCH information from ICT channels by mothers. Those who participated in these studies confirmed improved access to accurate MCH information and access to health care.

The result of the study revealed that the mothers had positive perception of the use of mobile phones. This was because the use of phones helped them to overcome the challenge of distance or access to health workers. Mobile phones allowed the mothers to access MCH information without having to travel long distance. This is probably the reason for its acceptance by the mothers, as indicated in similar studies (Onoriode, Otunomeruke, Ofuogbu, Mohammed, & Anyanti, 2012; Oyeyemi, 2012). The respondents also indicated a strong preference for radio (See Table 1). This implies that the mothers were more comfortable receiving MCH information by phone or radio. Corroborating this, Parmar's (2010) submission with respect to India stresses the importance of radio as a widely accepted channel that has been in use for decades to deliver information on women's health. This finding was also in tandem with Bowen (2010) and Murthy (2010) in Ghana and Tanzania, respectively. But it differed from what was found in a study (Castle et al., 2011) in the northern part of Nigeria; the study indicated a negative disposition by the sampled mothers to the use of mobile phones for receiving MCH information owing to poor ICT skills. Although the findings from this study confirmed the popularity of mobile phone for disseminating MCH information, some of the mothers reported being more comfortable with the radio.

In this study, the mothers expressed a negative perception towards the Internet (Not Useful, 67.7%). This finding differed from results obtained by authors in the developed countries (Childbirth Connection, 2012; Sayakhot & Carolan‐Olah, 2016). Earlier studies indicated a high level of acceptance of the Internet by mothers. In this study, only the mothers in Imo State expressed a positive perception towards the Internet. This may be due to better access to Internet services in the urban area in Imo state, unlike other facilities located in the rural areas where the level of ICT infrastructure was far below what is obtained in Imo state. Therefore, better Internet connection services are needed to improve the effectiveness of ICT interventions in Nigeria.

The findings from this study flag a caution signal to developers of e‐health projects to consider the perception of ICT and the information being disseminated to a target group when designing these projects. It also implies that the popularity of a particular ICT tool may not necessarily indicate its use by the target group, because the study participants also indicated a strong positive disposition to the use of radio probably due to its affordability and wide coverage (Parmar, 2010). This realisation may have informed Parmar (2010) and Musoke's (2002) submission that ICT based intervention for MCH in Africa should adopt both user centric and multitechnology approaches. That is, users’ approval of technologies should be sought and an approach that adopts more than one ICT tool should be considered when implementing e‐health projects. Therefore, health educators and e‐health project designers may need to study the target group to evolve effective e‐health projects for MCH care. In addition, information professionals developing MCH information products should adopt acceptable ICT tools; they should also present health tips in local languages or dialects to ensure the comprehension of the messages being disseminated.

## Conclusion

The mothers sampled had positive perception towards MCH information from ICT channels. This implies that mothers will use ICT to receive maternal health information and also adopt MCH information promoted through ICT once they are convinced that it will help to improve their health. To effectively promote MCH information through ICT, there is a need for information professionals and developers of ICT based projects to always put into consideration the perception of MCH mothers. The content of MCH information product also needs to be reviewed to enhance mothers’ understanding of the messages being disseminated. Future ICT based projects should adopt more than one ICT tool and more consideration should be given to the development of more MCH information products in local languages to ensure the effectiveness of the use of ICT for MCH information dissemination.

## Conflict of interest

The authors declare no conflict of interest.

## Funding

This research was partially funded by a fellowship award provided by the Consortium for Advanced Research Training in Africa (CARTA). CARTA has been funded by the Wellcome Trust (UK) (Grant No: 087547/Z/08/Z), the Department for International Development (DfID) under the Development Partnerships in Higher Education (Del‐ PHE), the Carnegie Corporation of New York (Grant No: B 8606), the Ford Foundation (Grant No: 1100–0399), Google.org (Grant No: 191994) Sida (Grant No: 54100029) and the Bill and Melinda Gates Foundation (Grant No: 51228).
